# Free energy profiles for unwrapping the outer superhelical turn of nucleosomal DNA

**DOI:** 10.1371/journal.pcbi.1006024

**Published:** 2018-03-05

**Authors:** Hidetoshi Kono, Shun Sakuraba, Hisashi Ishida

**Affiliations:** 1 Molecular Modeling and Simulation Group, Department of Quantum Beam Life Science, National Institutes for Quantum and Radiological Science and Technology, Umemidai, Kizugawa, Kyoto, Japan; 2 Graduate School of Frontier Sciences, The University of Tokyo, Kashiwanoha, Kashiwa, Chiba, Japan; National Institutes of Health, UNITED STATES

## Abstract

The eukaryotic genome is packaged into a nucleus in the form of chromatin. The fundamental structural unit of chromatin is a protein-DNA complex, the nucleosome, where 146 or 147 base pairs of DNA wrap 1.75 times around a histone core. To function in cellular processes, however, nucleosomal DNA must be unwrapped. Although this unwrapping has been experimentally investigated, details of the process at an atomic level are not yet well understood. Here, we used molecular dynamics simulation with an enhanced sampling method to calculate the free energy profiles for unwrapping the outer superhelical turn of nucleosomal DNA. A free energy change of about 11.5 kcal/mol for the unwrapping agrees well with values obtained in single molecule experiments. This simulation revealed a variety of conformational states, indicating there are many potential paths to outer superhelicdal turn unwrapping, but the dominant path is likely asymmetric. At one end of the DNA, the first five bps unwrap, after which a second five bps unwrap at the same end with no increase in free energy. The unwrapping then starts at the other end of the DNA, where 10 bps are unwrapped. During further unwrapping of 15 bps, the unwrapping advances at one of the ends, after which the other end of the DNA unwraps to complete the unwrapping of the outer superhelical turn. These results provide insight into the construction, disruption, and repositioning of nucleosomes, which are continuously ongoing during cellular processes.

## Author summary

The eukaryotic genome is compactly packaged into the cell nucleus. Within the nucleus, DNA forms protein-DNA complexes called nucleosomes. The nucleosome is the fundamental structural unit of chromatin. It is composed of a histone octamer around which DNA wraps about twice. During cellular processes such as DNA transcription, replication and repair, the DNA must be unwrapped from the structure. This unwrapping has been investigated experimentally, but details of the process at the atomic level are not yet well understood. We carried out molecular dynamics simulations using an enhanced sampling method to obtain free energy profiles for unwrapping the outer superhelical turn of the nucleosomal DNA. Our analysis shows that some energy cost is required to unwrap the first 5 bps at one end of the DNA, after which a second 5 bps unwrap with no increase in free energy. The unwrapping then proceeds at the other end of the DNA for up to 10 bps. In this way, DNA unwrapping occurs asymmetrically from one end. These findings provide insight into how DNA dynamics are related to gene regulation.

## Introduction

The nucleosome is the fundamental structural unit of chromatin and is composed of histone proteins and DNA. Its crystal structure revealed that within the nucleosome, 146 or 147 bps of DNA wrap 1.75 times around an octameric histone core [1]. However, for proper gene function, such as transcription, duplication and repair, this nucleosomal DNA must be unwrapped, at least partially.

Single-molecule experiments showed that the energetics of protein-DNA interactions depend to varying degrees on the protein-DNA contact positions. FRET experiments[2–6] showed that in the absence of salt, the outer superhelical turn of DNA (hereinafter referred to as “outer DNA”) within the nucleosome repeats spontaneously and repeatedly unwrapping and rewrapping. Rewrapping is about 5 times faster than unwrrapping, which is within about 10 to 50 ms [7, 8]. Single-molecule experiments in which the two ends of the DNA in a nucleosomal array [2, 5] or individual nucleosome [6] are mechanically pulled in opposite directions demonstrated that the outer DNA is more easily unwrapped than the inner super-helical turn. Later, after performing a DNA unzipping experiment, Wang’s group reported the energetics of histone-DNA interactions within the nucleosome [4]. This single molecule experiment showed that distinct histone-DNA interactions occur at intervals of about five bps, indicating that the two phosphate backbones of double-stranded DNA interact independently with the histones. It is difficult, however, to determine at the atomic level details such as which interaction is lost first during unwrapping and then second and so on.

All-atom molecular dynamics (MD) simulations have provided detailed information about the dynamics of the nucleosome [9–16]. Ettig et al. were the first to carry out all-atom MD simulations of the nucleosome to investigate unwrapping energetics [17] and Gribkova et al. calculated histone-DNA binding energies with a concept similar to this study [18]. Consistent with the unzipping experiment, Ettig et al. observed a 5-bp periodicity. However, because of the limited amount of time that could be computationally processed, a large force was imposed on the DNA during the unwrapping process, which caused unrealistic distortion of the DNA and histones.

Coarse-grained MD is an attractive method because it enables processing time spans not achievable in all-atom simulations using current state-of-the-art computer systems. Kenzaki and Takada performed a coarse-grained MD simulation that captured residue-level structural details, and exhibited spontaneous unwrapping and rewrapping of the outer DNA [19]. In addition, their simulation showed that the unwrapping is mostly asymmetric, with one end unwrapping while the other end remains wrapped. Using a similar coarse-grained model, Lequieu et al. calculated the tension-dependent free energy surface and found that the contributions of histone tails to DNA stability differed between the H3/H4 and H2A/B tails [20]. Whereas H3/H4 contributes more to the stability of the outer DNA, H2A/B stabilizes the inner DNA. The results of these simulations are consistent with the experimental findings [2–6]. However, coarse-grained MD requires adjustable parameters for the target of interest and is still less reliable in dynamics than all-atom simulations with an explicit solvent model due to the nature of the simplification.

Here, we carried out MD simulations with an enhanced sampling method to obtain conformational ensembles. We report the free energy profiles for unwrapping the outer-turn of nucleosomal DNA. The obtained profiles based on the ensembles show that unwrapping of the outer-turn costs about 11.5 kcal/mol. Comparison of the numbers of unwrapped base pairs at the two ends of the DNA shows that the unwrapping occurs asymmetrically, though the nucleosome has a pseudo-symmetric structure and a symmetric DNA sequence.

## Results

**Fig 1:**
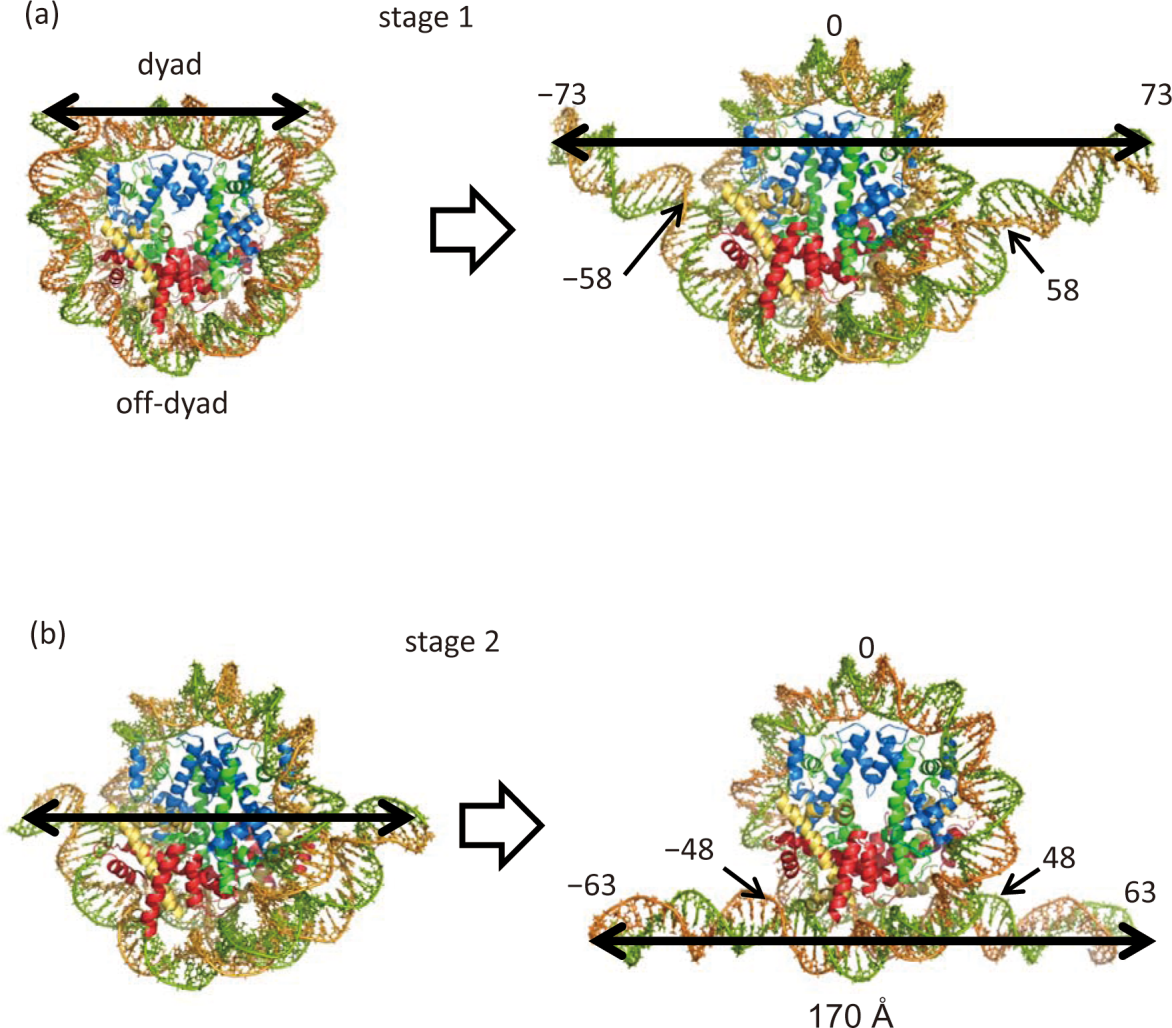
The initial and final conformations in the outer DNA unwrapping simulations. Shown are DNA numbering and unwrapped DNA regions in stages 1 (a) and 2 (b). H3: blue, H4: green, H2A: yellow and H2B: red.

We carried out simulations of unwrapping outer superhelical turn DNA (outer DNA) to obtain the free energy profiles. Here, we define “outer DNA” as the DNA regions extending from one end to a position 10 bps from the off-dyad position: −73 to −48 and +48 to +73 (see Fig 1, stage 1 plus stage 2). Crystal structures show that the histone core has 14 separate DNA binding domains [1]. In this study, we unwrapped 25 bps of DNA from each end (50 bps in total), which corresponds to a loss of interaction with four DNA binding domains, two from each of end of the DNA. In these unwrapping simulations, we divided the unwrapping into two stages: stage 1, −73 to −58 and stage 2, −63 to −48. The initial structures for stage 2 were prepared by deleting 10 bps from each end of the DNA. This reduced the computational cost by making the simulation size smaller.

### Convergence of adaptively biased molecular dynamics (ABMD) simulations and free energy calculations during stages 1 and 2

In ABMD simulations, a biasing energy is added to the potential energy to destabilize the recently visited positions in the reaction coordinate. A “flooding time” τ determines how fast the current position is destabilized. A larger drop of biasing energy given by a smaller flooding time can save computational time because energy wells along the reaction coordinate are filled faster. However, a short flooding time, for instance τ = 1 ps, often corrupted the dsDNA structure of the nucleosomal DNA. We tested three τ values, 1, 10 and 100 ps, with 100 walkers and found that 10 ps was mostly acceptable from a structural point of view. However, to further ensure proper dsDNA structure was maintained, we set τ to 100 ps. Note that the flooding time, τ value has to be determined considering the total number of walkers used in ABMD simulation because the total energy dropped during the simulation is proportional to the product of 1/τ and the total number of walkers. With this τ value, ABMD simulations with 100 walkers were carried out for 15 ns (1.5 ms in total), until the dropped energy converged at every point over the entire range of the reaction coordinate (S1A and S2A Figs). One can see that the walkers moved back and forth on the reaction coordinate (S1B and S2B Figs) until the biasing energy dropped every 1 ns became uniformly in the considered region on the reaction coordinate.

To then refine the free energy, umbrella sampling was carried out with 71 bins 2 Å in width from 40 to 180 Å in stage 1 and with 26 bins 2 Å in width from 125 to175 Å in stage 2. The umbrella sampling was not carried out for the region from 24 to 40 Å or beyond 180 Å in stage 1, or for the region beyond 175 Å in stage 2 because abnormal DNA distortions and base pair breaks were observed (S3A and S3B Fig) at the excluded end-to-end distances.

**Fig 2:**
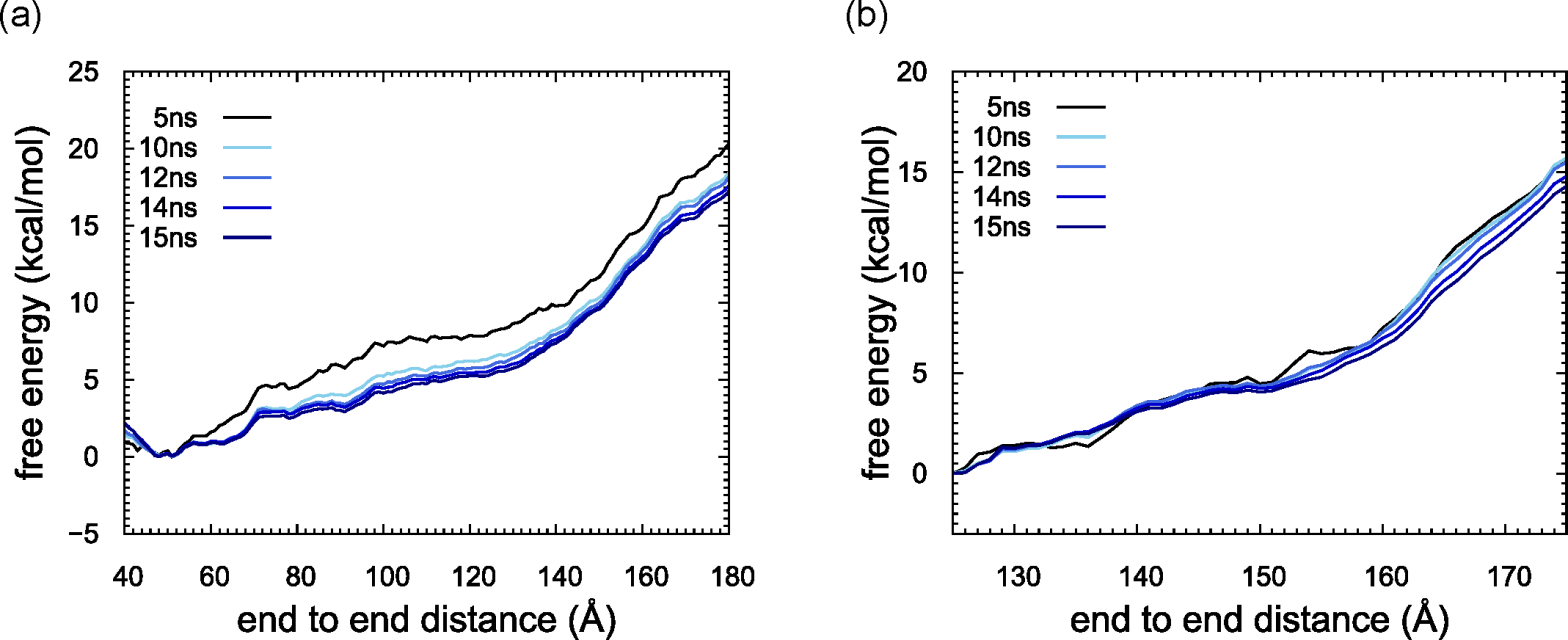
Free energy profiles plotted as a function of DNA end-to-end distance. Free energies were calculated using the ensembles obtained with umbrella sampling durations of 5, 10, 12, 14 and 15 ns from black to blue. (a) stage 1. (b) stage 2.

We show that free energy changed along the reaction coordinate in Fig 2. We first checked the convergence of the free energy. The energy curves calculated using different umbrella sampling times with WHAM mostly overlapped with each other after 10 ns. In both stages 1 and 2, free energy was finally obtained using a sampling duration of 15 ns (1.14 ms and 0.465 ms in total, respectively).

### Free energy as a function of the number of unwrapped base pairs

**Fig 3:**
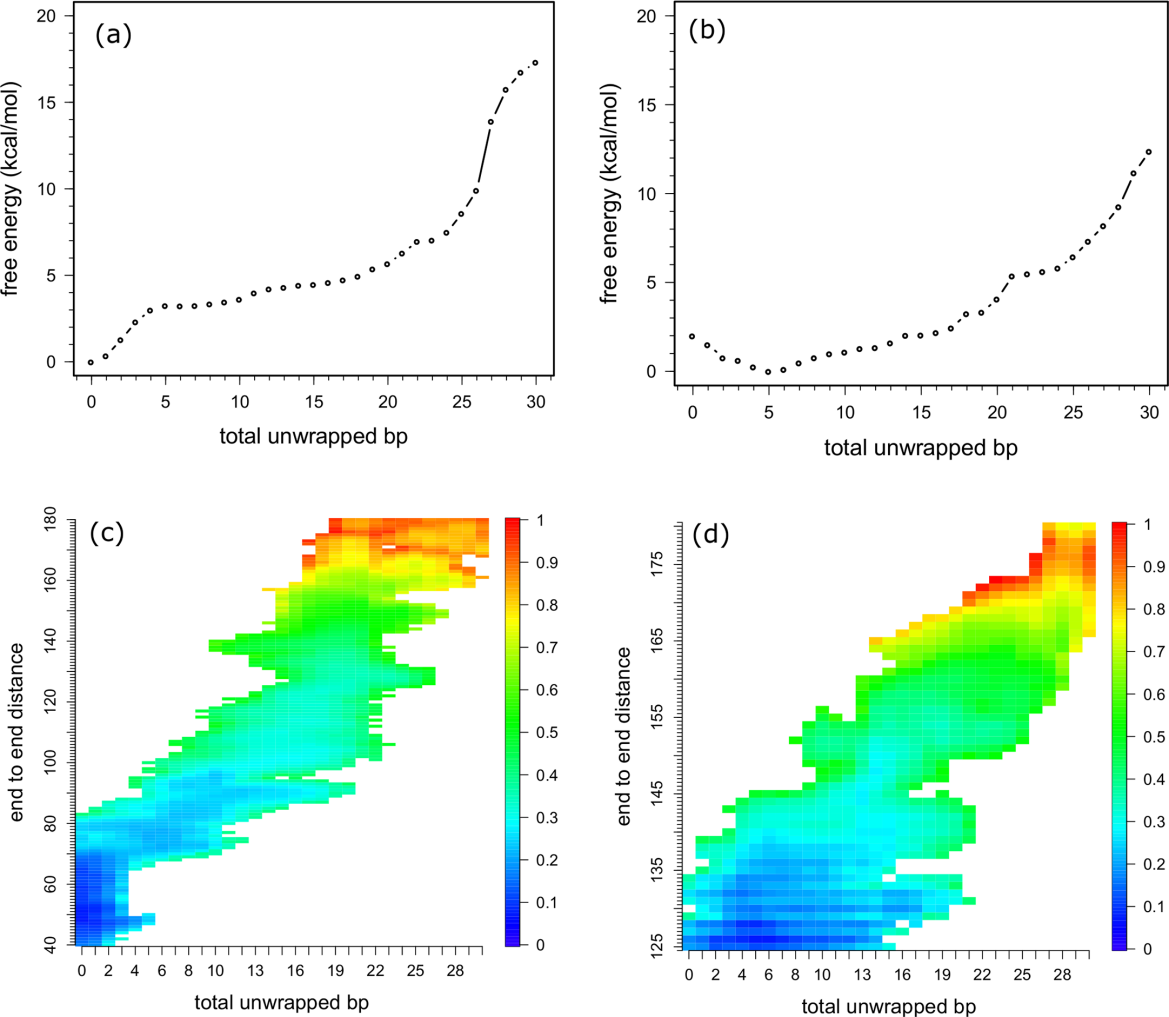
**Free energy profiles plotted as a function of the total number of unwrapped bps (a) and (b), or as a function of the total number of unwrapped bps and end-to-end distance (c) and (d).** (a) and (c) stage 1. (b) and (d) stage 2. Colors in (c) and (d) denote the relative free energy in each stage.

To interpret the unwrapping process from a structural viewpoint, we calculated the free energy as a function of the total number of unwrapped base pairs using Eqs 6 and 10 (Fig 3A and 3B), and as a function of the end to end distance and total number of the unwrapped base pairs (Fig 3C and 3D). We defined unwrapped base pairs as those in which the center of the base pair deviated more than 4 Å from the center of the histone core in the reference structure, where DNA was fully wrapped. As the reference structure, we used the initial structure used for ABMD subjected to energy minimization and a 1 ns long relaxation run without constraints.

During stage 1, the free energy profiles indicated that it costs 3.5 kcal/mol to unwrap the first five bps, but the next five bps were unwrapped with only a small increase in free energy. Thereafter, about 0.2 kcal/mol/bp were required for the unwrapping until after a total of 20 bps were unwrapped, at which point the free energy reached 5.5 kcal/mol. As shown in Fig 2, the slope of the free energy sharply increased when the end-to-end distance reached 130 Å, which corresponds to unwrapping about 20 bps (Fig 3C). As shown in Fig 3C, end-to-end distance takes a wide range of values (90 to 180 Å) at 20 bps in total unwrapped bp, and a free energy minimum is located around 130 Å, at which the slope of free energy curve sharply increases in Fig 2. Since unwrapping simulation in stage 2 starts with 20 bps shorter DNA than that of stage 1, the slope of the free energy curve at the beginning in stage 2 was expected to be similar to that of stage 1 after 130 Å. However, the slope of stage 1 was nearly two times larger than that of stage 2. Then, to check DNA conformation, we calculated base pair and base step parameters of 10 bps from both ends for the 15 ns trajectories of the umbrella samplings using X3DNA[21] and tabulated them in S1 to S4 Tables for stage 1 and S5 to S8 for stage 2. In the end DNA regions, there are only AT, CG, GC and TA steps out of the possible 16 steps. These tables clearly show that DNA conformations except for roll are realistic compared with values in references [22, 23]. However, “roll” of CG started to decrease and “rise” of TA started to increase at 130 Å in end-to-end distance in stage 1 (see S3 and S4 Tables), indicating that some of energy was used for DNA bending or stretching rather than for unwrapping. This indicates that a longer time would be necessary for relaxing the stress in the DNA bending and further unwrapping. Therefore, we did not consider data for unwrapping more than 20 bps.

During stage 2, the free energy decreased with unwrapping the first five bps, which corresponds to 20–25 bp in stage 1, probably because unfavorable stresses in DNA were relaxed. This is another reason we stopped the unwrapping analysis at 20 bp in stage 1. The energy then gradually increased until a total of 17 bps were unwrapped. There are two small gaps between 13 and 14 bps and between 17 and 21 bps. After those points, four bps were unwrapped from the histone core without cost. The free energy started to increase again at 25 bps, reaching 6 kcal/mol, after which we again observed a sharp increase in free energy. This position indeed corresponds to an end-to-end distance of about 160 Å (Fig 3D), which is consistent with a position where the slope showed a sharp increase in the free energy as a function of end-to-end distance (Fig 2B). As shown in Fig 3D, a minimum in free energy at 25 total unwrapped bp is around 160 Å in end-to-end distance. DNA conformation analyses shows that “roll” base step parameter of TA and AT steps started to deviate from the standard value and decrease, indicating DNA bending (S5 and S8 Tables).

### Lost interactions with histones and DNA

**Fig 4:**
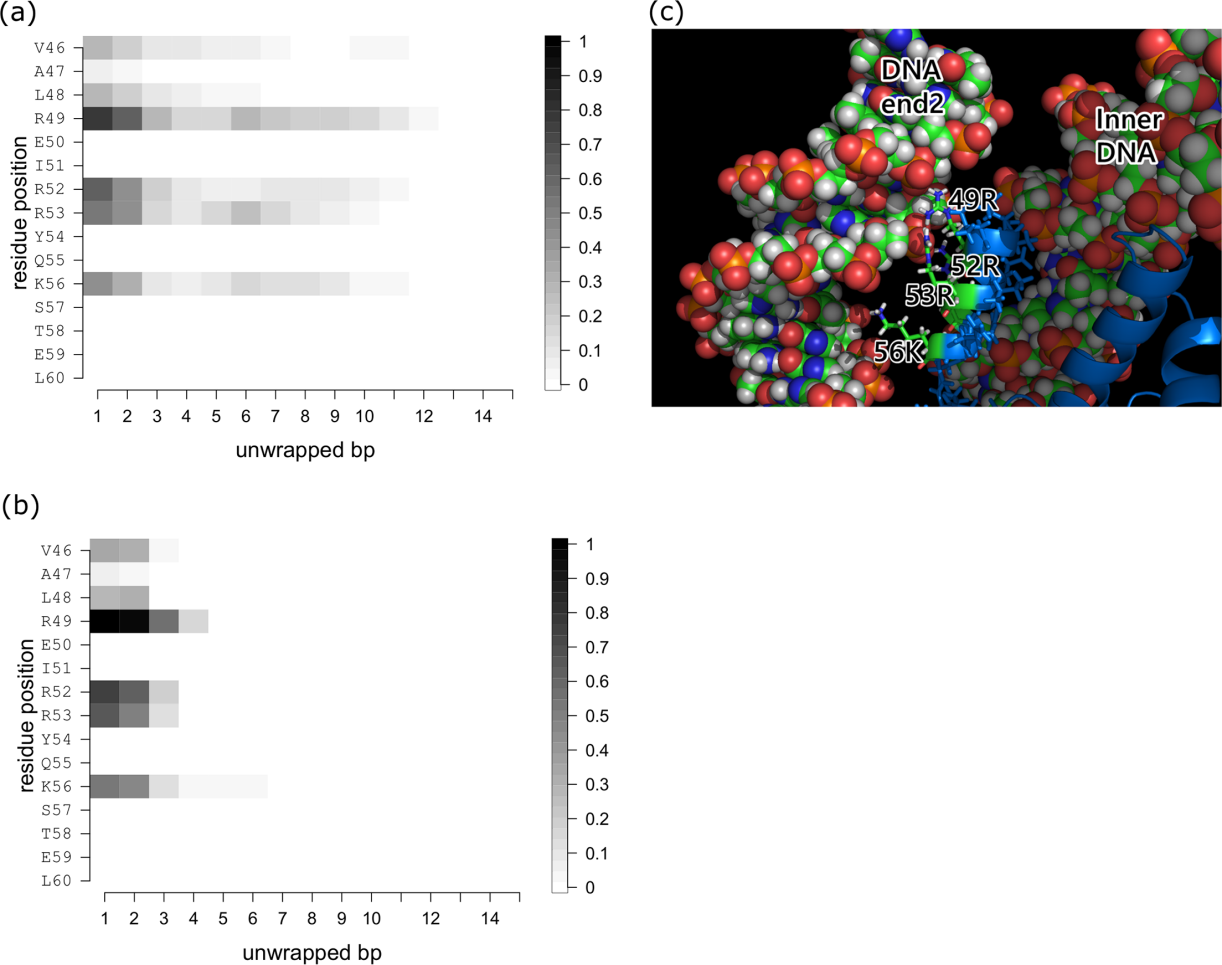
Histone-DNA contacts changing as a function of the number of unwrapped bps at one DNA end during stage 1. (a) H3a-DNA end2 contacts. (b) H3b-DNA end2 contacts. Plotted are the contact probabilities of each residue in the conformational ensemble. Contact is counted if at least one pair of atoms in the histone and DNA is within 4 Å of each other. (c) Close-up view of the H3-DNA end interface. Residues 46 to 60 of H3 are located between DNA end2 and the inner DNA. The DNA and residues 46 to 60 are shown as space filling and stick models, respectively. H3 is shown as a cartoon model and colored blue.

We calculated the residue-wise contact ratio between the protein and DNA as a function of the number of unwrapped base pairs from one DNA end (Figs 4 and S4).

#### Stage 1

During stage 1, we observed changes in protein-DNA contact only on H3 histones (Figs 4 and S3). Because the nucleosome core particle contains two copies of each histone and the DNA has two ends, we distinguish them with suffixes “a” and “b” for the histones and “1” and “2” for the first and second halves of the nucleosomal DNA, respectively. Overall, the contact profiles for the two copies were similar, suggesting the sampling was sufficient in this stage of the calculation.

Within the wrapped nucleosome core particle, residues 46 to 56 of both H3 histones, especially R49, R52, R53 and K56, interact with both ends of the DNA (Fig 4A and 4C). These residues all participate in formation of the αN helix, which is situated between the end and the inner-turn of the DNA. Upon unwrapping the first five bps, the H3 tails lose contact with the inner-turn of the DNA entirely (Fig 4B) and lose most of the contacts with the end of DNA. This indicates that the αN helix is dragged outward with the end of DNA, an effect that can be captured as the increase in free energy (Fig 3A and 3B). The backbones of the hydrophobic residues V46 and L48 also interact with the DNA (Fig 4A) in the wrapped conformation.

Because the αN helix of H3 moves with the end of the DNA, contact between H3 and the end of DNA is observed up to the time 10–11 bp are unwrapped at one of the DNA ends, though the contact ratio gradually decreases with the unwrapping (Fig 4A). During this process, the free energy increases slightly from 3 to 5 kcal/mol (Fig 3A).

#### Stage 2

**Fig 5:**
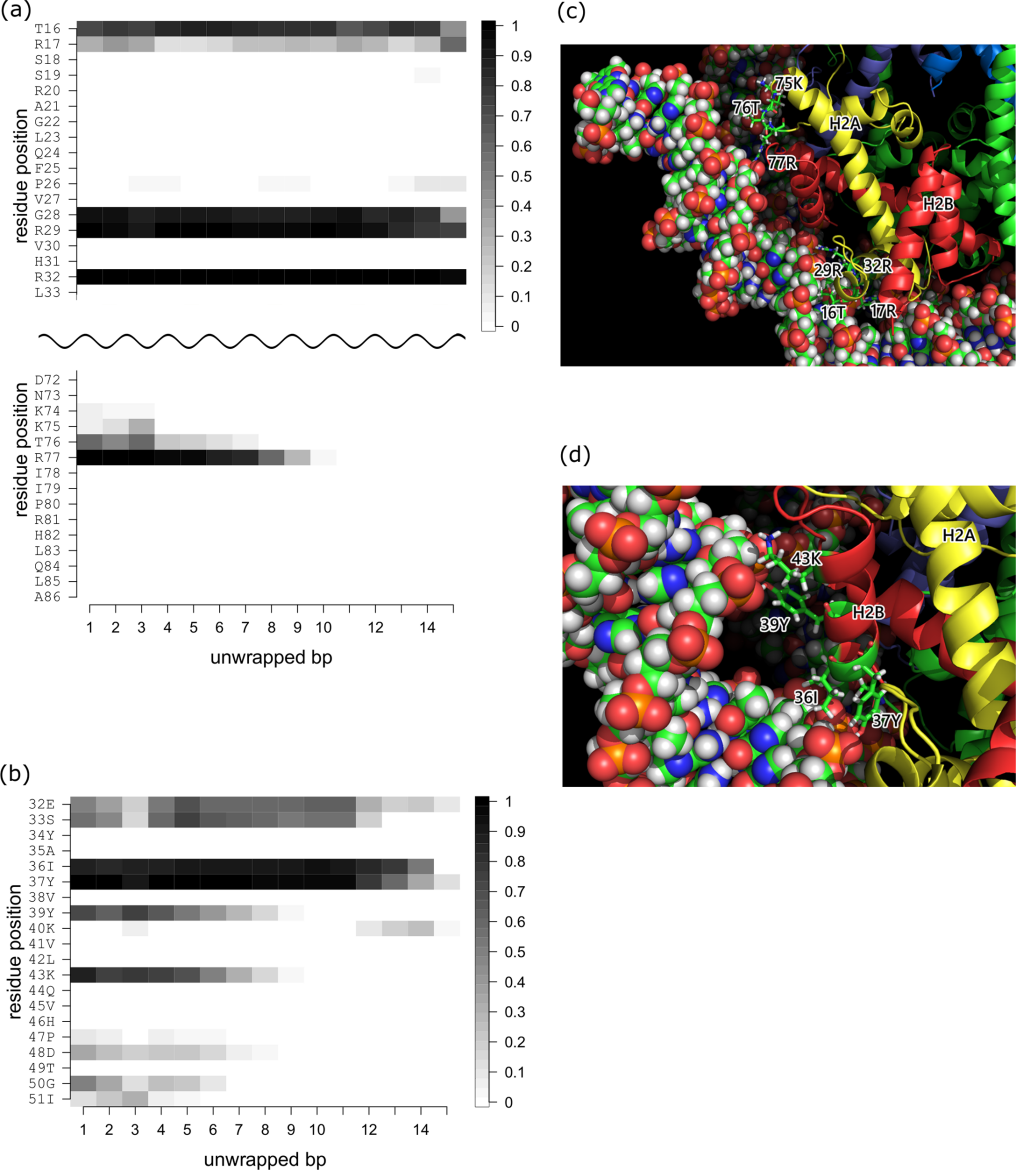
Histone-DNA contacts changing as a function of the number of unwrapped bps at one DNA end during stage 2. (a) H2Aa-DNA end2 contacts. (b) H2Ba-DNA end2 contacts. Plotted are the contact probabilities of each residue in the conformational ensemble. Contact is counted if at least one pair of atoms in the histone and DNA is within 4 Å of each other. (c) Close-up view of the H2Aa-DNA end2 interface. Residues 16 to 32 and 75 to 77 are shown as stick models. H2A and H2B are shown as cartoon models and are colored yellow and red, respectively. (d) Close-up view of the H2Ba-DNA end2 interface. The DNA and residues 36 to 43 are shown as space filling and stick models, respectively. H2A and H2B are shown as cartoon models and are colored yellow and red, respectively.

During stage 2, we observed changes in the protein-DNA contacts of H2A and H2B but not H3 or H4. Each of the two copies of H2A and H2B makes contact with only one end of the DNA. Again, the contact profiles for the two DNA ends were similar to one another, suggesting the sampling was sufficient in this stage’s calculation (Figs 5A and S5).

Within the starting conformation of H2A, T16, R17, G28, R29, R32 and residues 74 to 77 (KKTR) in the L2 loop make contact with the DNA (Fig 5C). During the unwrapping process, contact between the L2 loop and the DNA is lost as the first 10 bps are unwrapped, though the other contacts are maintained. Within H2B, E32 and S33 have moderate contacts with the DNA, while I36, Y37, Y39 and K43 have strong contacts. Among these, Y39 and K43 lose contact with DNA as the first 10 bps are unwrapped. At the beginning of the unwrapping in stage 2, moderate contacts between the DNA and P47 and D48, G50 and I51 are lost.

The loss of contacts is well correlated with the free energy curve shown in Fig 3B. There are small gaps in the free energy between the unwrapping of 13 and 14 bps and between 17 and 21 bps. A detailed structural analysis showed that the first gap corresponds to the loss of contacts between the DNA and T76 of H2A and D48, G50, and S51 of H2B, while the second gap corresponds to the loss of contact between the DNA and R77 of H2A and K43 in the α1 helix of H2B (Figs 5B and 5D and S5). Interestingly, R77 of H2A maintains contact with the DNA longer than any other residue, even though it is located close to the DNA end. The simulation shows that as the DNA is unwrapped, R77 is dragged out due to its strong electrostatic interaction with the DNA (Figs 5A and S5).

### On a way that DNA is unwrapped

**Fig 6:**
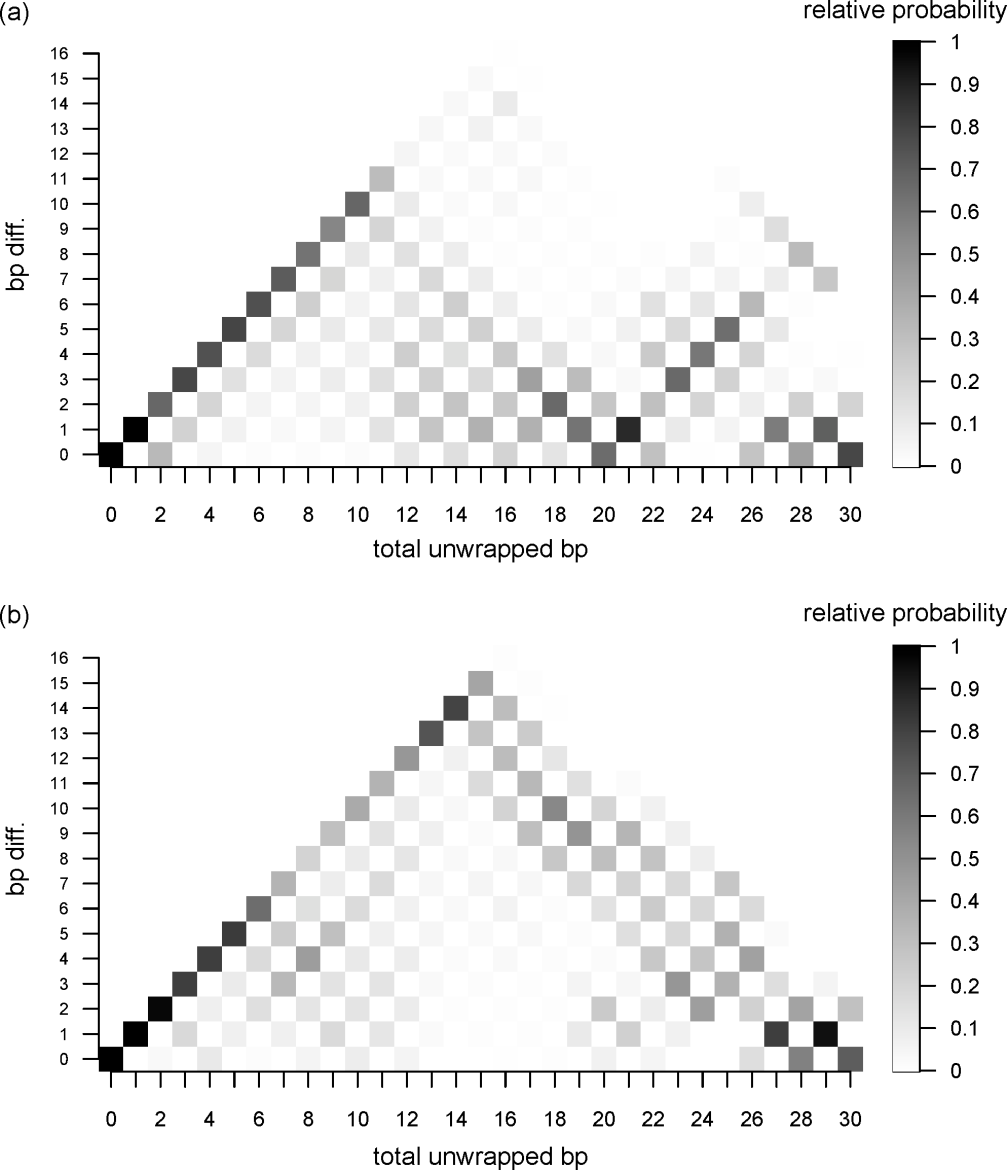
Differences in unwrapped bps between the two DNA ends. **The differences are plotted as a function of the total number of unwrapped bps.** The bp differences are normalized to the number of conformations with the same total number of unwrapped bps. (a) stage 1. (b) stage 2.

To understand the process of unwrapping of the outer DNA, we counted the number of unwrapped bps at each end and plotted the differences between them (Fig 6). In the figure, differences in the number of unwrapped bps are normalized to the number of conformations with the same total number of unwrapped bps to see the probability of the unwrapped states. The figure shows “asymmetric unwrapping” of the DNA, though there are many unwrapping paths. In stage 1, only one end of DNA is unwrapped first; the other end does not start to unwrap until the total number of unwrapped bps reaches 10. In fact, there is a small gap in the free energy curve at the point where 11 bps are unwrapped (Fig 3A). This can be interpreted as indicating that 10 bps are unwrapped at one end of the DNA before the other end starts to unwrap. When a total of 20 bps are unwrapped, most of the conformations have 10 bps unwrapped at each DNA end.

This asymmetric unwrapping could be due to the initial conformations. To check this possibility, we calculated the root mean square deviation (rmsd) between the initial conformations of the two DNA endpoints using heavy atoms on each of the 100 structures for stage 1. In the calculation, considered were 15 bps from the end of the DNA and a part of the histone core close to the DNA (13 residues from H3, 10 residues from H2A and 19 residues from H2B). The averaged rmsd value and standard deviation over 100 initial structures for stage 1 of ABMD which were obtained 100 ns long MD simulation are 2.70 Å and 0.65 Å, respectively. When only the DNA is considered, the corresponding values are 2.80 Å and 0.58 Å, respectively. These rmsd values are comparable with fluctuation of P atoms observed in the 100 ns MD simulation of the canonical nucleosome which maintained both DNA ends wrapped around the histone [12]. Thus, we consider that the initial conformations still maintain a pseudo-symmetric structure and the asymmetric unwrapping comes from the nature of stochastic process.

During stage 2, the asymmetry of the unwrapping was clearly observed as unwrapping at one end advanced up to 15 bps before the other end started to unwrap. Ultimately, both ends of the DNA were evenly unwrapped after unwrapping a total of 30 bps (50 bps, a total of stages 1 and 2).

**Fig 7:**
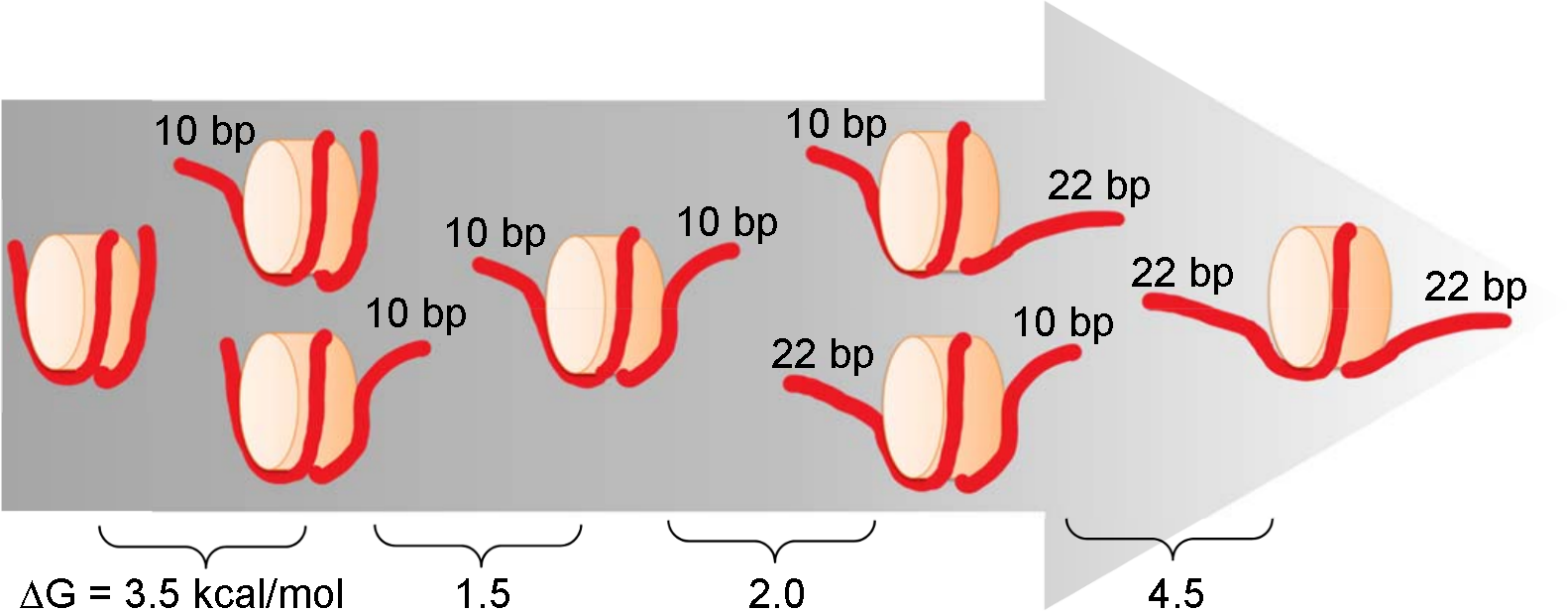
Schematic view of the dominant pathway to the unwrapping of outer DNA with estimated changes in free energy.

The scheme illustrated in Fig 7 shows a dominant path for unwrapping, though we observed a variety of unwrapped states. This asymmetric unwrapping indicates that interaction between nucleosomal DNA and the histone core is strengthened when either end of DNA is unwrapped. It is likely that increasing fluctuation at one end of the DNA promotes further unwrapping.

## Discussion

### On conformation sampling

We carried out simulations of DNA unwrapping from nucleosomes to obtain the free energy profiles. Nucleosomes have a pseudo-symmetric structure, and we used a symmetric DNA sequence. Consequently, there are two sets of interactions, as nucleosomes contain two copies for each of histones and two DNA ends. For instance, H3a-end2 and H3b-end1 contacts are expected to be very similar to one another. In fact, the contact profile of H3a and DNA end2 (Fig 4A) is very similar to that of H3b and DNA end1 (S4A Fig). This was also true for interactions between other histones and DNA, and demonstrates that the sampling in this study was sufficient to analyze these data.

### Free energy for outer DNA unwrapping

We considered free energy for the outer DNA unwrapping in two stages. In each stage, we observed unusual DNA stretch at longer end-to-end distances in ABMD simulations and therefore excluding those regions before the umbrella samplings. After checking the DNA conformations obtained by the umbrella samplings, we estimated the free energy cost of unwrapping a total of 44 bps, which corresponds to nearly the entire outer DNA, to be about 11.5 kcal/mol (5.5 kcal/mol in stage 1 and 6 kcal/mol in stage 2). The full unwrapping free energy has been estimated about 40 kcal/mol using an integrating model of a coarse-grain and fine-grain models[24] or a theoretical model [25]. The calculated cost of 11.5 kcal/mol for unwrapping 44 bps in this study is about a quarter of the cost for the full unwrapping, indicating the obtained value is quite reasonable compared with the reported values. The corresponding free energy value obtained by experiments ranges about 5 to 12 kcal/mol [2, 6, 26, 27], which likely depends on interpretation of the experimental results.

Because DNA unwrapping starts at one of the DNA ends (Fig 6), gaps in the free energy curves are well explained by the loss of interaction between the DNA and histones. However, there is a discrepancy between our model and a previous model [28]. Forties et al. proposed a quantitative theoretical model of DNA unwrapping and disassociation from the histone octamer [28]. By calibrating the model to a high-resolution nucleosome unzipping experiment [4], they successfully explained the differences in the rate of nucleosome disassembly caused by hMSH2-hMSH6 between two DNA sequences and between histone modification states. In their model, free energy increases stepwise as unwrapping advances, which is consistent with our findings. However, they found that the cost to unwrap 20 bps was about 1 k_B_T, which is 10 times smaller than our calculated value. Most likely, this discrepancy arises from the following differences. First, unwrapping and rewrapping rates are highly dependent on the salt concentration [3, 29, 30]. Second, in the simulation, we counted the number of unwrapped bps from both DNA ends. In the unzipping experiment, by contrast, the model building always occurred from one DNA end. Third, the definition of unwrapping is context dependent. They assumed that the number of broken base pairs equals the number of base pairs unwrapped from the nucleosome; however, it is quite possible that there is a gap in time between the unwrapping and the dissociation, which would lead to the observed small free energy for unwrapping 20 bps.

### Key residues in the DNA interaction

In an alanine mutation experiment, Ferreira et al. showed that each residue within the H3 αN helix revealed different aspects of nucleosome dynamics [31]. We found that the measured effects on DNA wrapping (see S1 Table in the reference) are well correlated with the contact ratio between the protein and DNA (Fig 4A). For example, R49A caused the largest decrease in the FRET ratio between the two DNA ends, followed by R52 and R53. Mutation of V46, L48 or K56 had a moderate effect. This trend is in good agreement with the contact ratios observed in the present study and in our earlier MD study [12], which indicates these residues are directly involved in protein-DNA interaction to stabilize the nucleosome. Interestingly, alanine mutation of L48 or Q55, which have little or no direct contact with DNA, destabilized histone octamer formation [31]. Most likely, these mutations destabilize the H3 αN helix causing favorable protein-DNA interactions to be lost. Thus, these residues are well conserved among eukaryotes.

During stage 2, we observed that T76 and R77 of H2A and Y39 and K43 of H2B gradually lose direct hydrogen bonds to the DNA. Consequently, the free energy curve showed a more gradual increase during the unwrapping process than during stage 1. However, most interactions with H2A and H2B were well maintained in stage 2 as shown in S3C and S3D Fig. Thus, the unwrapping process advances with a subtle increase in free energy. Interesting, the residues (R29, R32, R77 of H2A and Y37, K43 G50 and I51 of H2B) which are in contact with DNA as well as H2A or H2B in the form of the H2A-H2B heterodimer are well conserved among eukaryotes [32, 33] though H2A and H2B histones are the most sequence variable among all histones.

### Effect of histone tail removal on nucleosome stability

It should be noted that all simulations in this study were carried out on nucleosomes whose histone tails were truncated because our preliminary calculations showed that positively charged histone tails interact with the negatively charged DNA backbone, and sampled conformations are highly dependent on the initial conformations. This treatment has a possibility of underestimating the unwrapping free energy of mono-nucleosome though the tails have more important role in inter-nucleosome interaction as discussed below.

Histone tails are known to mediate interactions between nucleosomes, enabling them to fold into the chromatin solenoid [34], or they may mediate nucleosome assembly [35–38]. In particular, H4 tails probably play the most important role in inter-nucleosome interaction. All atom MD simulations by Langwoski’s group showed that H4K16 acetylation drastically reduces H4 tail-acidic patch of the stacked nucleosome and increases the tail conformational diversity [39]. Ishida & Kono computationally show that the diversity of nucleosome-nucleosome stacking depends on the way of H4 tail interactions [40]. These results indicate the impact of post-translational modification to chromatin conformation.

At mono-nucleosome level, what is the role of tails? At least in terms of thermodynamic view, thermal stability of the entire nucleosome changes little with or without the tails at mono-nucleosome level. Reported theoretical [24] and experimental [41, 42] works show that the tails have little effect (at most 1.1 kcal/mol) on thermodynamic stability of the entire nucleosome, but the tails can affect the partial unwrapping kinetics of the DNA. For instance, in a single molecule study, Brower-Toland et al. showed that tail truncation reduces the overall affinity of histones for outer DNA [43]. Interestingly, in SAXS and FRET experiments, Andersen et al. showed that truncation of the H3 or H4 tail has opposite effects. In their FRET studies, whereas H3 truncation induces dissociation of DNA, H4 truncation stabilizes the wrapped conformation to a greater degree than the intact nucleosome [44]. When Biswas et al. used all-atom MD simulations to examine nucleosome dynamics with and without the histone tails, they found that tail truncation destabilized the histone core [11]. Consistent with that finding, using an implicit solvent model, Erler et al. found that attractive interactions between positively charged Lys and Arg residues in the histone tails and negatively charged phosphate groups in the DNA stabilized the nucleosome structure [45]. In addition, a MD simulation carried out by one of the authors showed that the H3 N-terminal tail and H2A C-terminal tail both interact with linker DNA [13], and that the latter stabilizes the closed form of the linker DNA. Finally, biochemical [46] and MD [16, 47–49] studies showed that acetylation of the tails increases the α-helix content, and one of studies indicated that acetylation of the H3 tail induces DNA dissociation from the histone core [47], which is consistent with a single molecule FRET experiment [29, 50].

Here, we summarize the unwrapping outer DNA process. The calculated free energy profile suggests that about 11.5 kcal/mol are required for unwrapping of the first 22 bps from both DNA ends, which is consistent with the previously estimated values. The number of unwrapping bp differences between two DNA ends clearly exhibits DNA dissociation process is stochastic and asymmetrically advances up to the first 10 bps unwrapping, then unwrapping of another 10 bps follows at the other end. This process occurs once more to complete the unwrapping of the outer superhelical turn.

## Methods

### Atomic models

We used a crystal structure of a nucleosome, which we obtained from the Protein Data Bank (code:1kx5)[51]. Because histone tails are intrinsically disordered regions, they were truncated to accelerate nucleosomal DNA unwrapping and to ensure that it reached an equilibrium state within the limited simulation time. Residues 46 to 132 in H3, 25 to the C-terminal in H4, 16 to 114 in H2A, and 32 to 121 in H2B were used for MD simulations. The effect of the histone tails, which is likely to be of significance, will be the subject of a future investigation.

### Adaptively biased MD combined with the multiple walker method

To evaluate the free energy for unwrapping nucleosomal DNA from the histone core, the adaptively biased molecular dynamics (ABMD) method [52] combined with the multiple walker method [53] was implemented using an in-house software program, SCUBA [54–58]. To minimize the computational cost, we separated the outer DNA unwrapping process into two stages. Stage 1 involved −73 to −58 at DNA end1 (73 to 58 at DNA end2), while stage 2 involved −63 to −48 at DNA end1 (63 to 48 at DNA end2) (Fig 1). The reaction coordinate *d* was defined as the DNA end to end distance, or the distance between two phosphate atoms of T73 in chain I and of T73 in chain J according to the residue numbering in PDB code 1kx5. To make the water box smaller to save computational cost, stage 2 started after shortening the unwrapped DNA by 10 bps from each end of walker 70 in the umbrella sampling (end-to-end distance is 180 Ån of stage 1 (see below on the umbrella sampling). Finally, simulations were carried out with a fixed box size of 150 × 150 × 150 Å^3^ box in stage 1 or 158 × 223 × 160 Å^3^ in stage 2.

In the ABMD with the multiple walker method, we ran 100 independent MD simulations with one shared biased potential, which enabled sampling of different positions along the reaction coordinate [52]. The initial structures of the replicas used for stage 1 were snapshots saved every 1 ns from the trajectories in an earlier 100-ns canonical MD simulation [12]. Similarly, the initial structures used for stage 2 were prepared with a canonical 10-ns simulation starting with the final conformation of the most unwrapped replica in stage 1, and snapshots were saved every 100 ps from the trajectory. Force fields used were AMBER ff99SB[59], ff99bsc0[60] and ff99ions08[61], and the TIP3P[62] water model was used. The system was solvated in the 120 mM solution of NaCl, and the excess negative charges of the nucleosome complex were neutralized by the excess number of sodium ions. The time step was 2 fs, and all bonds containing hydrogen were frozen with the SHAKE algorithm [63, 64]; the temperature was regulated at 300 K with Langevin dynamics with a collision frequency of 2.0 ps^−1^ for each atom [65]; electrostatic interactions were calculated with the particle-particle particle-mesh (PPPM) method [66, 67]; and van der Waals interactions were calculated with a cutoff of 9.0 Å. In all simulations, the center of mass of the histone protein was constrained by a harmonic function with a spring constant of 10.0 kcal/mol to exclude the effect of translational movement of the histone protein.

We carried out ABMD simulations until the biased potential became mostly flat over the range of the reaction coordinate. The range of the reaction coordinate was set at 20 to 200 Å in stage1 and 110 to 250 Å in stage 2. To keep all walkers within the predefined range, a harmonic potential with a force constant of 10.0 kcal/mol was applied at *d* = 25 and 195 Å in stage 1 and 115 and 245 Å in stage 2. The resolution of the reaction coordinate *Δd* was set at 1.0 Åat 1.0 resolution of the reactfree-energy profile τ was set at 100 ps, and the ABMD biasing potential was updated every step. For instance, in stage 1 an energy of 13.88 kcal/mol per p was added to the biasing potential for 1 ns with τ = 100 ps using 100 replicas. In total, 1.5 μs of ABMD simulations (15 ns per replica) for stages 1 and 2 were carried out. In ABMD and the following umbrella sampling calculations, we assigned 48 nodes (each node has12 cores) for each replica. All calculations were carried out using K supercomputer.

### Umbrella sampling and the free-energy profile using WHAM

Theoretically, if *τ* is large enough and the width of the kernel used in the ABMD is small enough, they converge towards the free energy. However, when using a *τ* with a given time length, the free-energy landscape, or negative of the biasing potential, fluctuates during the ABMD simulation [55]. For that reason, umbrella sampling was done to enhance equilibrium sampling. In the umbrella sampling, the reaction coordinate was divided into 71 windows with a width of 2 Å, which covered 40 to 180 Å in stage 1. In stage 2, it was divided into 26 windows with a width of 2 Å, covering 125 to 175 Å. We prepared the initial conformations for umbrella sampling as follows. First, we sorted 100 walkers according to end-to-end distance in ABMD. Then, out of the 100 walkers, we picked up walkers whose end to end distance was closest to $d_{i}^{\mathrm{fix}}$ where $d_{i}^{\mathrm{fix}}$ = 40 Å + 2.0 Å × i (i = 0, 1, 2, …, 70) in stage 1. Likewise, for stage 2, we picked up walkers according to $d_{i}^{\mathrm{fix}}$ = 125 Å + 2.0 Å × i (i = 0, 1, 2, …, 25). The umbrella potential for each window is a harmonic function with a force constant of 0.2 kcal/(mol Å^2^). Note that the sampled conformations in ABMD at less than 40 Å and more than 180 Å on the reaction coordinate in stage1 and more than 175 Å in stage 2 were discarded because their DNA structures were clearly corrupted or highly distorted (S3 Fig).

The weighted histogram analysis method (WHAM) [68, 69] was used to refine the free-energy landscape from the sampled trajectories in the umbrella sampling simulations. With the WHAM approach, the unbiased probability distribution *P*(**R**) is calculated from the biased probability distribution of the sampled coordinates as:

$$P\left(R\right)=\sum_{i=1}^{N_{win}}n_{i}\;\left(R\right)P_{i}^{\left(b\right)}\;\left(R\right)\times {\left[\sum_{j=1}^{N_{win}}n_{j}\;\left(R\right)\exp\left(\left[F_{j}-V_{j}\left(R\right)\right]/k_{B}T\right)\right]}^{-1},$$ (1)

where **R** is the atomic coordinates, *N*_*win*_ is the number of windows, *n*_*i*_(**R**) is the number of data points in the *i*-th window, *P*_*i*_^*(b)*^(**R**) is a biased probability from the raw data obtained in the umbrella sampling simulation, *V*_*j*_(**R**) is the biasing potential in the *j*-th window, *k*_*B*_ is the Boltzmann constant, and *T* is the constant temperature. In this study, *V*_*j*_(**R**) was selected to be the sum of a harmonic potential and the ABMD biasing potential in the final stage of the ABMD simulation, which has the form:

$$V_{i}\left(R\right)=k_{i}{\left(d\left(R\right)-d_{i}^{\mathrm{fix}}\right)}^{2}+U_{abmd},$$ (2)

where *d*(**R**) is the distance between the centers of mass of the two terminal nucleotides of the nucleosomal DNA. $d_{i}^{\mathrm{fix}}$ is a fixed distance to maintain *d*(**R**) within the range of 40 to 180 Å with intervals of 2 Å (*i* = 0,…, *N*_*win*_ = 70) in stage 1 and 125 to 175 Å with intervals of 2 Å (*i* = 0,…, *N*_*win*_ = 25) in stage 2. *k*_*i*_ is an arbitrary harmonic force constant, which was set at 0.2 kcal/mol/Å^2^. The umbrella sampling simulation was carried out for 15 ns. The conformation of the nucleosome for the analysis was stored every 1 ps.

The coefficient *F*_j_ is defined by:

$$F_{j}=-k_{B}T\ln\left\{\sum_{\mathrm{windows}}^{}P\left(R\right)\exp\left(\left[-V_{j}\left(R\right)\right]/k_{B}T\right)\right\},\;(j=1,\ldots N_{win})$$ (3)

where the summation includes all the coordinates of **R**, which were sampled in any windows. By iterating Eqs (1) and (3) to achieve self-consistency (using a tolerance of 10^−8^), the relative free energy *F*(**R**) at a given **R** is obtained as:

$$F\left(R\right)=-k_{B}T\ln P(R).$$ (4)

To visualize the free-energy profile, the dimension of **R** in Eq (4) was reduced to 1 or 2 dimensions by defining an appropriate coordinate (reaction coordinate). The reaction coordinate could be simply selected to be *d*(**R**); however, *d*(**R**) may not be a good reaction coordinate, as it is not a direct indicator of how far the unwrapping of the nucleosomal DNA has progressed from the initial state. Instead, the total number of base pairs unwrapped from the histone core would be a good indicator for interpreting the DNA unwrapping. Consequently, the first reaction coordinate, *R*_*1*_, was defined as *d*(**R**), and the second reaction coordinate, *R*_*2*_, was defined as the total number of unwrapped base pairs. Unwrapping was defined when the center of mass of a base pair was shifted outward by more than 4 Å from the crystal structure. The probability of the trajectories on *R*_*2*_, *P*(*R*_*2*_), can be written as:

$$P(R_{2})=\int P(R')\delta (R_{2}-R_{2}^{'}(R'))dR',$$ (5)

where *δ(X)* is the Dirac delta-function, and $R_{2}^{'}=R_{2}^{'}(R)$. The free-energy profile in 1 dimension has the same form as Eq (4):

$$F\left(R_{2}\right)=-k_{B}T\ln P(R_{2}).$$ (6)

To describe the changes in a physical quantity, *A*, such as the distance between atoms along *R*_*2*_, the averaged quantity at *R*_*2*_, *Ā*(*R*_*2*_), is calculated by weighing the unbiased probability of the quantity *A*(*R*) as:

$$\bar{A}\left(R_{2}\right)=\frac{\int A(R\prime )P(R\prime )\delta (R_{2}-R_{2}^{'}(R\prime ))dR\prime }{P(R_{2})}.$$ (7)

The root mean square deviation (rmsd) from *Ā*(*R*_*2*_) is

$$\sqrt{\sigma^{2}\left(R_{2}\right)}=\sqrt{\bar{A^{2}}\left(R_{2}\right)-\bar{A}{\left(R_{2}\right)}^{2}}$$ (8)

The probability and the 2-dimensional free-energy landscape with regard to *R*_*1*_ and *R*_*2*_, *P*(*R*_*1*_, *R*_*2*_) and *F*(*R*_*1*_, *R*_*2*_), respectively, are expressed as:

$$P(R_{1},R_{2})=\int P(R\prime )\delta (R_{1}-R_{1}^{'}(R'))\delta (R_{2}-R_{2}^{'}(R')dR\prime ,$$ (9)

$$F\left(R_{1},R_{2}\right)=-k_{B}T\ln P(R_{1},R_{2}).$$ (10)

## Supporting information

S1 Fig Dropped energy per 1 ns along the reaction coordinate (energy per 1 ns) in stage 1. (b) Changes in the positions of 100 walkers along the reaction coordinate against time in stage 1.
(TIF)

S2 Fig Dropped energy per 1 ns along the reaction coordinate (energy per 1 ns) in stage 1. (b) Changes in the positions of 100 walkers along the reaction coordinate against time in stage 2.
(TIF)

S3 Fig DNA end-to-end distance plotted as a function of distance between two phosphorus atoms in base pairs of 100 walkers in ABMD simulations. (a) Plot at every 1 ns from 15 to 20 ns of ABMD in stage 1. Symbols denote base pair positions: red box for base pair at 72; blue star at 71; black cross at 62. (b) Plot at every 1 ns from 10 to 15 ns of ABMD in stage 2. Symbols denote base pair positions: red box for at 62; blue star at 61; black cross at 52.
(TIF)

S4 Fig Histone-DNA contacts changing as a function of the number of unwrapped bps at one DNA end during stage 1. (a) H3a-DNA end2 contacts. (b) H3b-DNA end1 contacts. Plotted are the contact probabilities of each residue in the conformational ensemble. A contact is counted if at least one pair of atoms in the histone and DNA is within 4 Å of each other.
(TIF)

S5 Fig Histone-DNA contacts changing as a function of the number of unwrapped bps at one DNA end during stage 2. (a) H2Ab-DNA end1 contacts. (b) H2Bb-DNA end1 contacts. Plotted are the contact probabilities of each residue in the conformational ensemble. A contact is counted if at least one pair of atoms in the histone and DNA is within 4 Å of each other.
(TIF)

S1 Table Base pair and base step parameters of AT steps in two DNA ends (10 bps from each) in stage 1. DNA conformations in the umbrella sampling (15 ns) were analyzed using X3DNA^21^.
(XLSX)

S2 Table Base pair and base step parameters of CG steps in two DNA ends (10 bps from each) in stage 1. DNA conformations in the umbrella sampling (15 ns) were analyzed using X3DNA^21^.
(XLSX)

S3 Table Base pair and base step parameters of GC steps in two DNA ends (10 bps from each) in stage 1. DNA conformations in the umbrella sampling (15 ns) were analyzed using X3DNA^21^.
(XLSX)

S4 Table Base pair and base step parameters of TA steps in two DNA ends (10 bps from each) in stage 1. DNA conformations in the umbrella sampling (15 ns) were analyzed using X3DNA^21^.
(XLSX)

S5 Table Base pair and base step parameters of AT steps in two DNA ends (10 bps from each) in stage 2. DNA conformations in the umbrella sampling (15 ns) were analyzed using X3DNA^21^.
(XLSX)

S6 Table Base pair and base step parameters of CG steps in two DNA ends (10 bps from each) in stage 2. DNA conformations in the umbrella sampling (15 ns) were analyzed using X3DNA^21^.
(XLSX)

S7 Table Base pair and base step parameters of GC steps in two DNA ends (10 bps from each) in stage 2. DNA conformations in the umbrella sampling (15 ns) were analyzed using X3DNA^21^.
(XLSX)

S8 Table Base pair and base step parameters of TA steps in two DNA ends (10 bps from each) in stage 2. DNA conformations in the umbrella sampling (15 ns) were analyzed using X3DNA^21^.
(XLSX)
