# Correction: Free energy profiles for unwrapping the outer superhelical turn of nucleosomal DNA

**DOI:** 10.1371/journal.pcbi.1007439

**Published:** 2019-10-09

**Authors:** Hidetoshi Kono, Shun Sakuraba, Hisashi Ishida

S4 Fig is incorrect. The authors have provided a corrected version here.

Fig 4 and S4 Fig legends are incorrect. The authors have provided a corrected version here.

**Fig 4:**
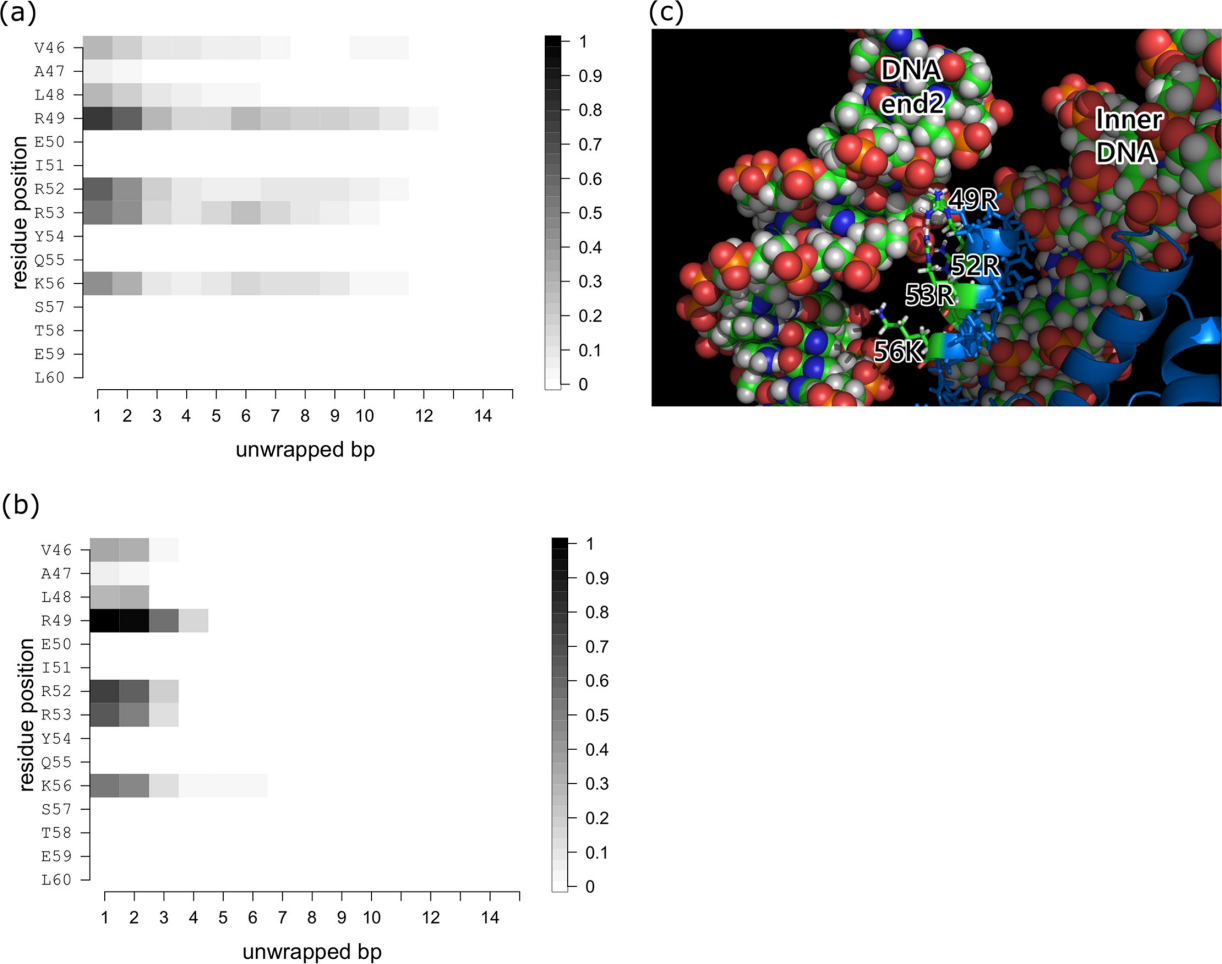
Histone-DNA contacts changing as a function of the number of unwrapped bps at one DNA end during stage 1. (a) H3a-DNA end2 contacts. (b) H3a-DNA end1 contacts. Plotted are the contact probabilities of each residue in the conformational ensemble. Contact is counted if at least one pair of atoms in the histone and DNA is within 4 Å of each other. (c) Close-up view of the H3-DNA end interface. Residues 46 to 60 of H3 are located between DNA end2 and the inner DNA. TheDNA and residues 46 to 60 are shown as space filling and stick models, respectively. H3 is shown as a cartoon model and colored blue.

While revising the manuscript, the Authors replaced figures showing interactions of H3 histone with DNA but did not change the legends. Here, the Authors corrected the legends of Fig 4(B) and S4A and S4B Fig and provided the correct figure for S4B Fig. The correction does not affect their conclusion that nucleosomal DNA unwraps asymmetrically.

## Supporting information

S4 Fig Histone-DNA contacts changing as a function of the number of unwrapped bps at one DNA end during stage 1. (a) H3b-DNA end1 contacts. (b) H3b-DNA end2 contacts. Plotted are the contact probabilities of each residue in the conformational ensemble. A contact is counted if at least one pair of atoms in the histone and DNA is within 4 Å of each other.
(TIF)
