# Supplementary figures and images for: Free energy profiles for unwrapping the outer superhelical turn of nucleosomal DNA
Source: PLoS Comput Biol. 2018 Mar 5;14(3):e1006024. doi: 10.1371/journal.pcbi.1006024 (PMC5854429; doi:10.1371/journal.pcbi.1006024)

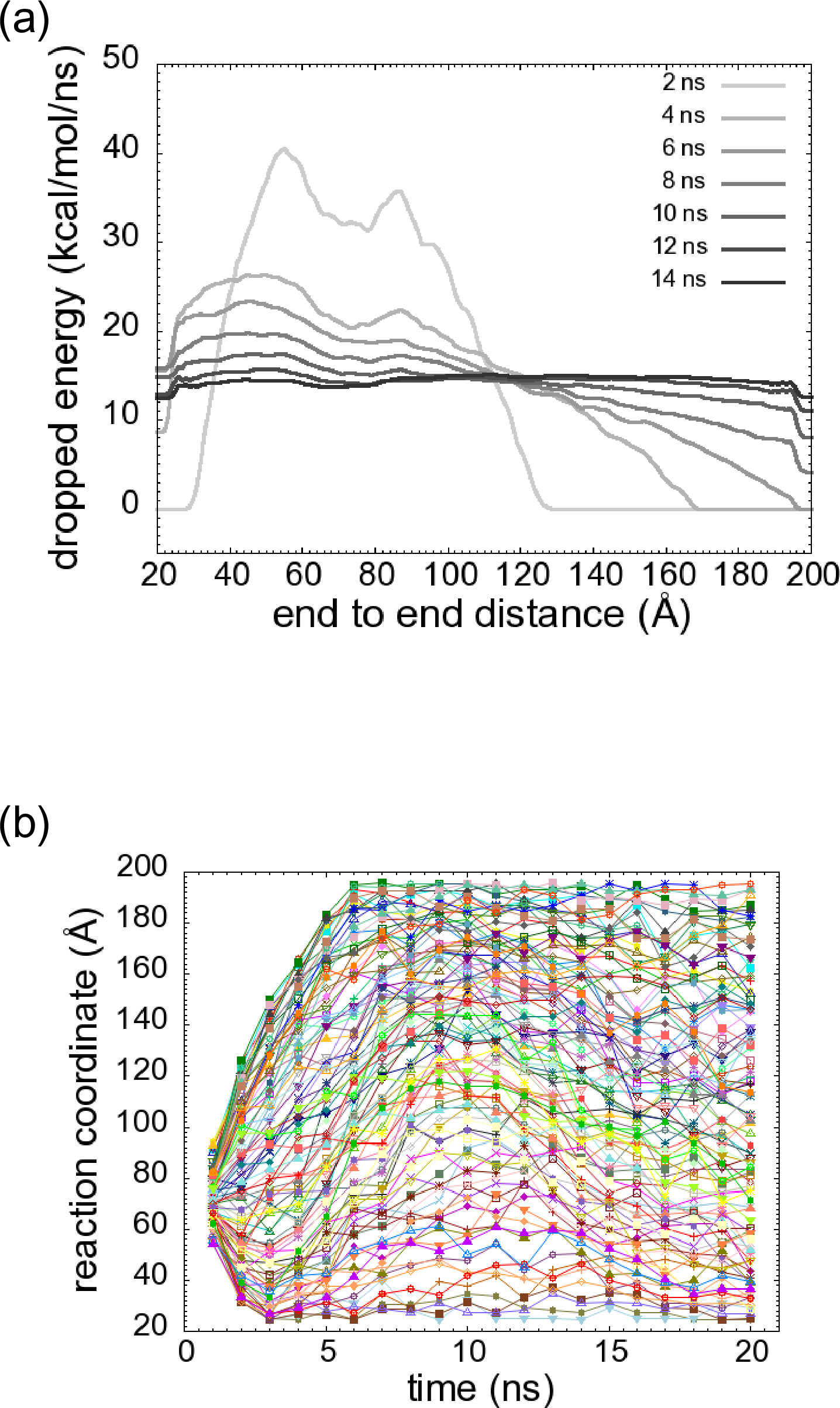

Supplement: S1 Fig — (b) Changes in the positions of 100 walkers along the reaction coordinate against time in stage 1. (TIF) [file pcbi.1006024.s001.tif]

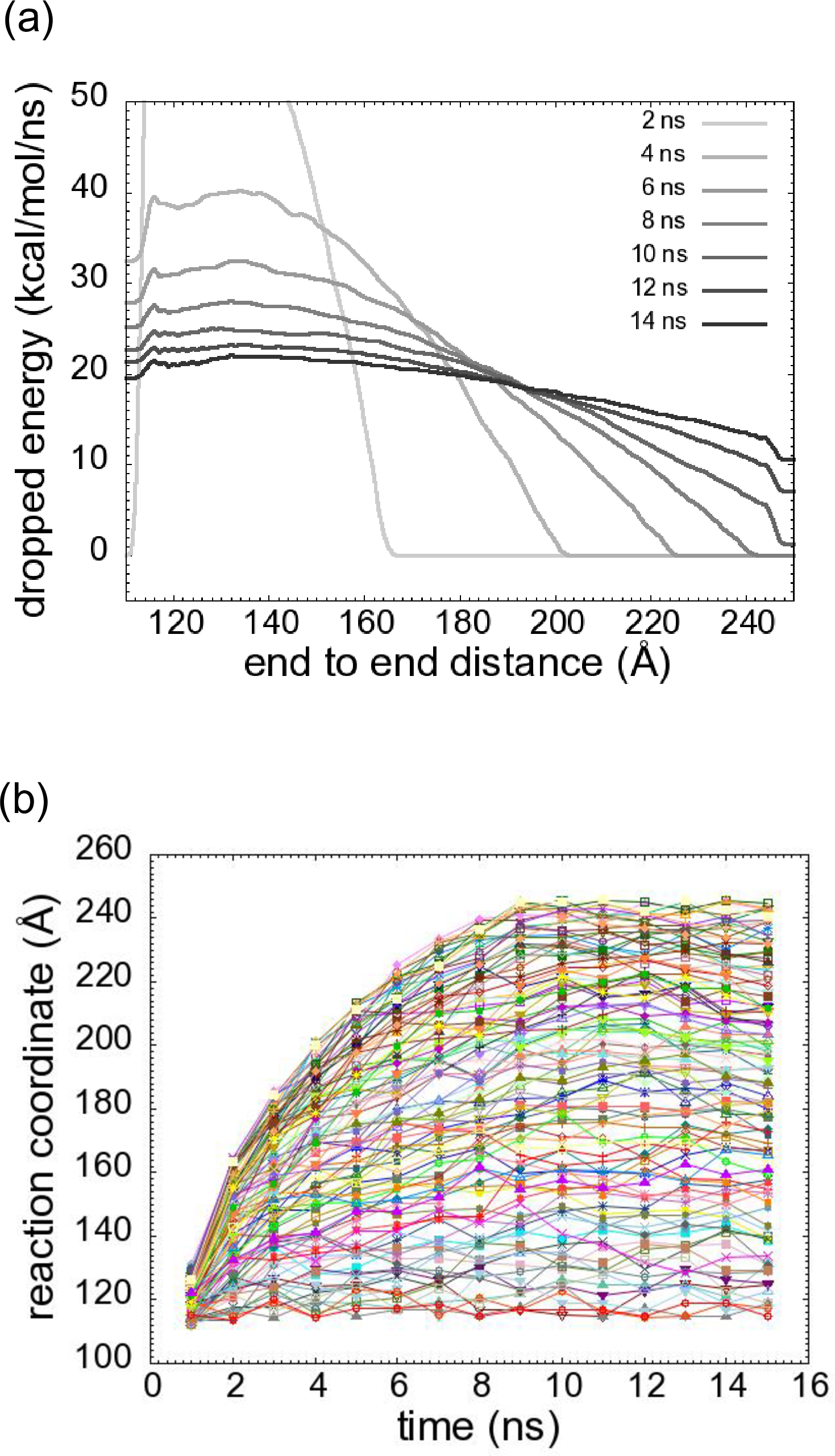

Supplement: S2 Fig — (b) Changes in the positions of 100 walkers along the reaction coordinate against time in stage 2. (TIF) [file pcbi.1006024.s002.tif]

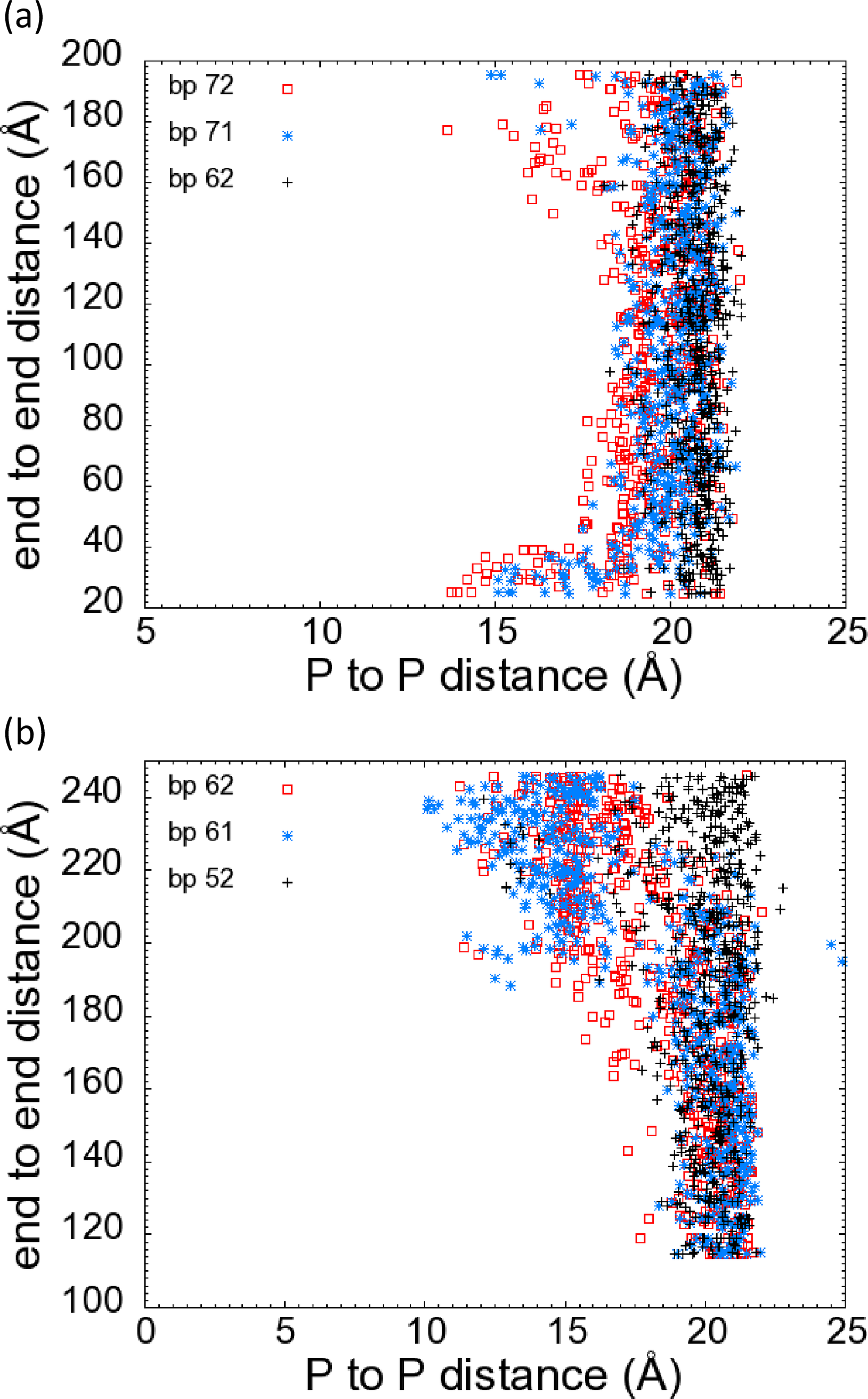

Supplement: S3 Fig — (a) Plot at every 1 ns from 15 to 20 ns of ABMD in stage 1. Symbols denote base pair positions: red box for base pair at 72; blue star at 71; black cross at 62. (b) Plot at every 1 ns from 10 to 15 ns of ABMD in stage 2. Symbols denote base pair positions: red box for at 62; blue star at 61; black cross at 52. (TIF) [file pcbi.1006024.s003.tif]

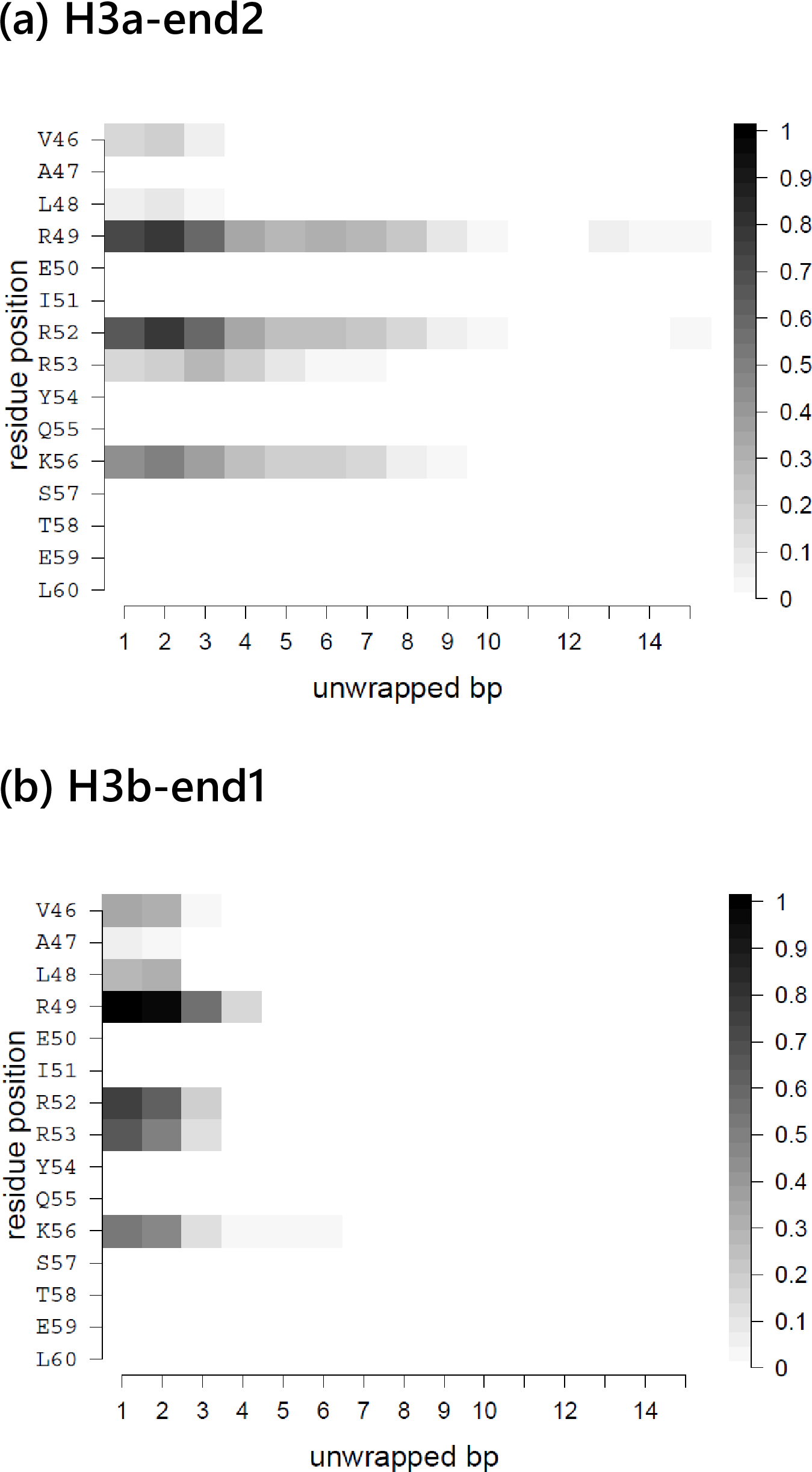

Supplement: S4 Fig — (a) H3a-DNA end2 contacts. (b) H3b-DNA end1 contacts. Plotted are the contact probabilities of each residue in the conformational ensemble. A contact is counted if at least one pair of atoms in the histone and DNA is within 4 Å of each other. (TIF) [file pcbi.1006024.s004.tif]

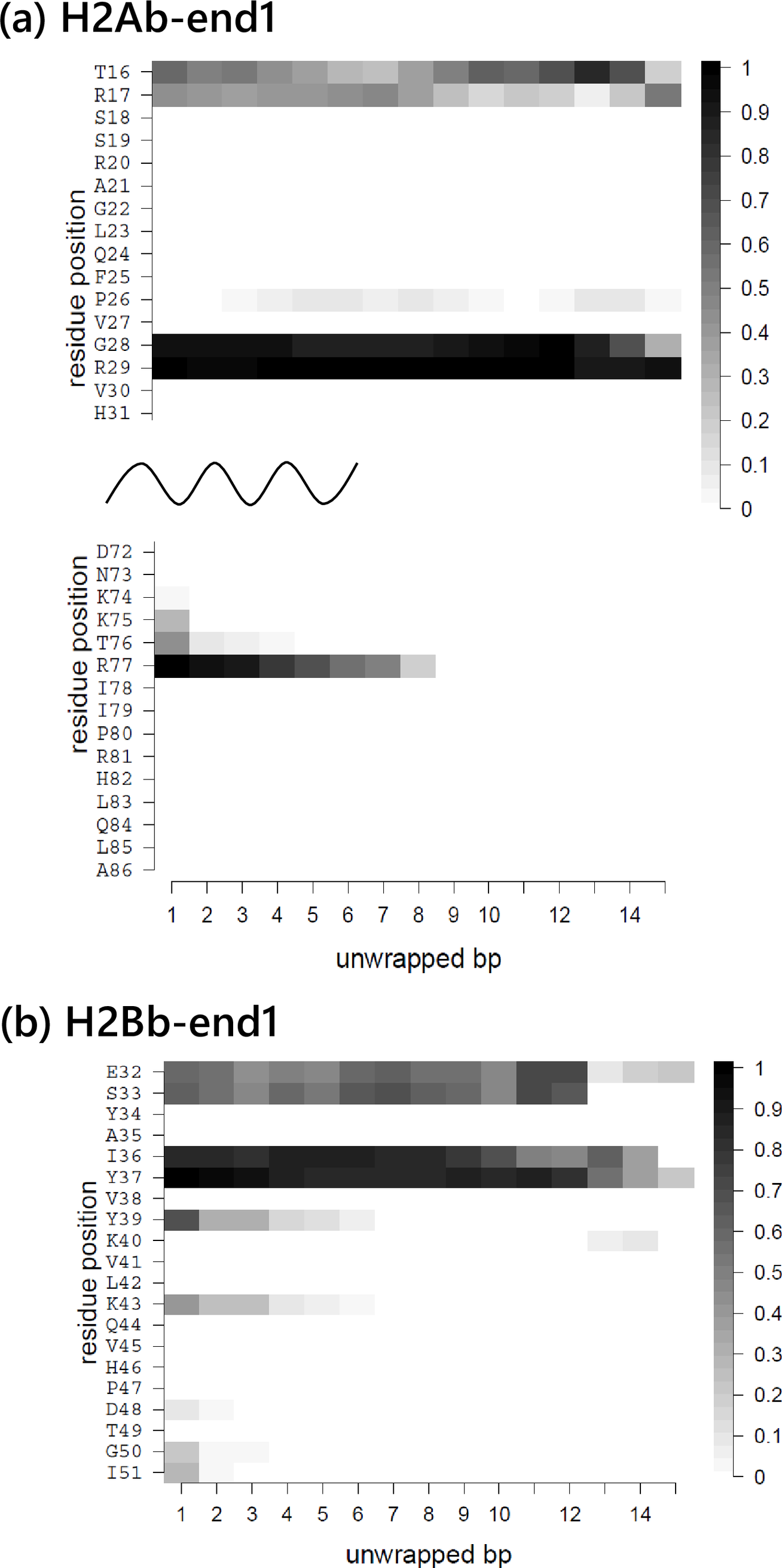

Supplement: S5 Fig — (a) H2Ab-DNA end1 contacts. (b) H2Bb-DNA end1 contacts. Plotted are the contact probabilities of each residue in the conformational ensemble. A contact is counted if at least one pair of atoms in the histone and DNA is within 4 Å of each other. (TIF) [file pcbi.1006024.s005.tif]
