# Supplementary figures and images for: Correction: Free energy profiles for unwrapping the outer superhelical turn of nucleosomal DNA
Source: PLoS Comput Biol. 2019 Oct 9;15(10):e1007439. doi: 10.1371/journal.pcbi.1007439 (PMC6785060; doi:10.1371/journal.pcbi.1007439)

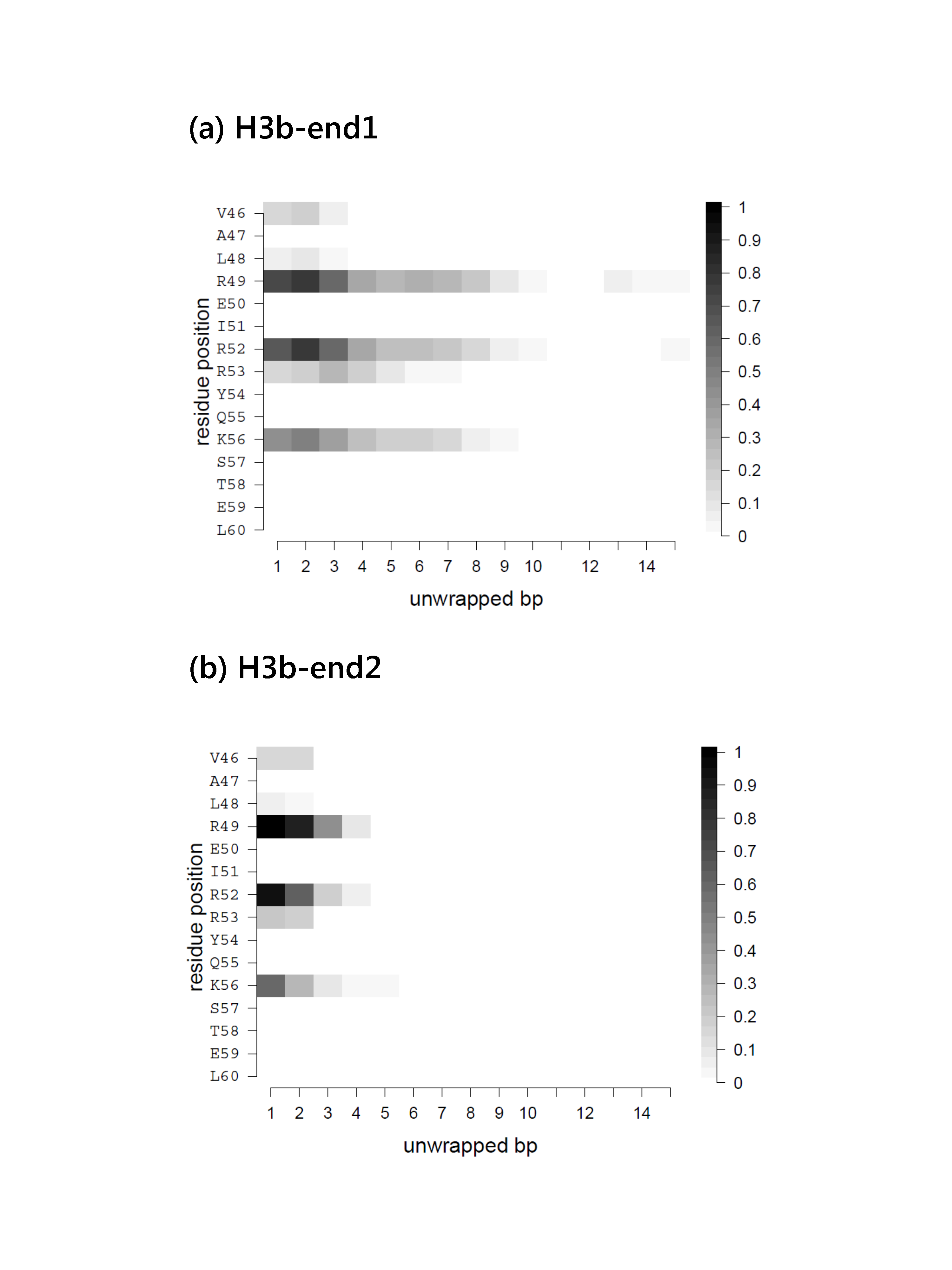

Supplement: S4 Fig — (a) H3b-DNA end1 contacts. (b) H3b-DNA end2 contacts. Plotted are the contact probabilities of each residue in the conformational ensemble. A contact is counted if at least one pair of atoms in the histone and DNA is within 4 Å of each other. (TIF) [file pcbi.1007439.s001.tif]
